# Metachronous Liver Metastasis from Alpha-Fetoprotein-Producing Gastric Cancer Successfully Treated with Capecitabine/Oxaliplatin Combination Chemotherapy

**DOI:** 10.1155/2022/2700394

**Published:** 2022-08-23

**Authors:** Soichi Furukawa, Takashi Kobayashi, Saori Shiono, Shuta Nishinakagawa

**Affiliations:** ^1^Department of Surgery, Tokyo Rosai Hospital, 4-13-21 Omori-Minami, Ohta-ku, Tokyo 143-0013, Japan; ^2^Department of Pathology, Tokyo Rosai Hospital, 4-13-21 Omori-Minami, Ohta-ku, Tokyo 143-0013, Japan; ^3^Department of Gastroenterology, Tokyo Rosai Hospital, 4-13-21 Omori-Minami, Ohta-ku, Tokyo 143-0013, Japan

## Abstract

A consensus regarding the treatment of recurrent alpha-fetoprotein-producing gastric carcinoma due to its rarity is lacking. We herein describe a case of such a carcinoma that was associated with metachronous liver metastasis. A 73-year-old man was referred for the surgical treatment of a type 2 gastric tumor that extended from the greater curvature of the gastric corpus to the pylorus. As no remote metastases were detected, the patient underwent open total gastrectomy with lymphadenectomy and Roux-en-Y reconstruction. Histopathological examination revealed regional lymph node metastasis and the invasion of the muscularis propria by a moderately differentiated adenocarcinoma. Immunostaining of the primary tumor was positive for alpha-fetoprotein and negative for human epidermal growth factor receptor 2. Serum alpha-fetoprotein levels decreased to within normal limits after eight courses of S-1 monotherapy; however, levels started to increase, and a hypovascular nodule in segment 5/6 of the liver was detected 3.5 years later. Serum alpha-fetoprotein levels returned to the normal range, and the tumor was undetectable after four courses of capecitabine and oxaliplatin therapy. No recurrence was detected at 1.5-year follow-up. This case demonstrates that combined capecitabine and oxaliplatin therapy can successfully treat metachronous liver metastasis from alpha-fetoprotein-producing gastric carcinoma.

## 1. Introduction

Alpha-fetoprotein- (AFP-) producing gastric carcinomas account for approximately 1.3–15% of all gastric cancers [1]. These carcinomas are associated with a higher rate of liver metastasis and poorer prognosis (5-year survival rate, 8–28%) than AFP-negative (ordinal) gastric carcinomas [1–4]. The clinicopathological characteristics and molecular mechanisms of AFP-producing gastric carcinomas are not fully understood due to their rarity. As a result, there is currently a lack of consensus for a standard therapeutic policy for AFP-producing gastric carcinomas, including cases with metachronous liver metastasis.

Several studies that have investigated AFP-producing gastric carcinomas over the past several decades have reported unsatisfactory outcomes following the curative resection of the primary tumor or liver metastasis. However, some reports have suggested that multimodal therapies, including surgery, other regional treatment, and chemotherapy, may be effective in improving patient prognosis [5]. We herein report a case of metachronous liver metastasis from AFP-producing gastric carcinoma after curative gastrectomy. This was successfully treated with capecitabine/oxaliplatin (CapeOX) combination chemotherapy.

## 2. Case Report

**Figure 1:**
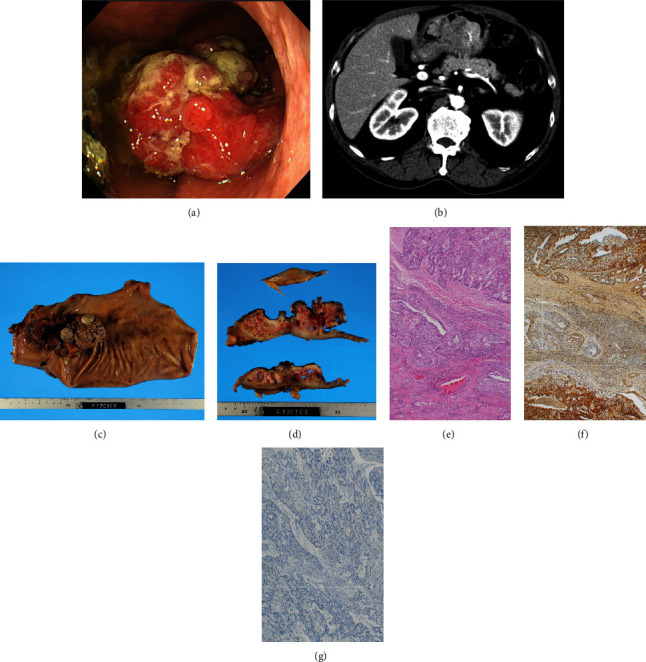
Preoperative findings and histopathological analysis of the resected specimen. Upper endoscopy revealing a semicircular type 2 tumor on the greater curvature of the gastric corpus that extends to the pylorus (a). Contrast-enhanced computed tomography depicting bulky thickening of the distal stomach wall, without invasion to the pancreas or ascites, lymph node or remote metastases, or liver tumors (hepatocellular carcinoma or metastatic liver tumor) (b). Open total gastrectomy, lymphadenectomy, and Roux-en-Y reconstruction were performed (c). The histopathological diagnosis was gastric cancer (pT2, N2, M0, stage IIB (Union for International Cancer Control TNM Classification of Malignant Tumors, 8th edition)) (d, e). Immunostaining of the primary tumor was positive for alpha-fetoprotein and negative for human epidermal growth factor receptor 2 (f, g, respectively).

A 73-year-old man with anemia was referred to the Department of Gastroenterology of our hospital. He had a positive fecal occult blood test and high serum AFP level, but no history of liver cirrhosis or hepatitis. Blood tests indicated hemoglobin, serum AFP, serum carcinoembryonic antigen, and serum carbohydrate antigen 19-9 levels of 8.4 g/dL (reference range, 13.5–18.0 g/dL), 207.7 ng/mL (reference range, 0.0–10.0 ng/mL), 45.8 ng/mL (reference range, 0.0–5.0 ng/mL), and 11 U/mL (reference range, 0–37 U/mL), respectively. Upper gastrointestinal endoscopy revealed an 11 × 5 cm semicircular Borrmann type 2 tumor on the greater curvature of the gastric corpus that extended to the pylorus (Figure 1(a)). A biopsy of the tumor showed a well to moderately differentiated adenocarcinoma. The wall of the distal stomach was thickened on contrast-enhanced computed tomography (CE-CT); no signs of ascites, lymph node metastasis, remote metastasis, or tumor invasion was noted in the pancreas (Figure 1(b)). CE-CT and gadolinium ethoxybenzyl-diethylenetriaminepentaacetic acid-enhanced magnetic resonance imaging (EOB-MRI) confirmed the absence of a liver tumor.

**Figure 2:**
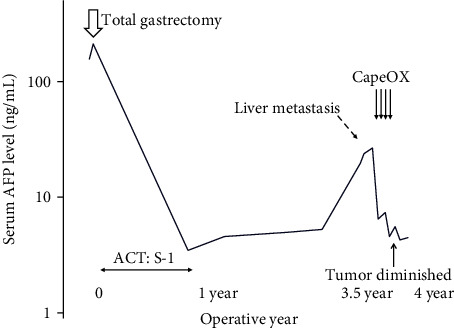
Chronological trend of serum alpha-fetoprotein levels serum alpha-fetoprotein (AFP) levels was elevated and abruptly decreased after curative gastrectomy. Metachronous liver metastasis and increases in serum AFP levels were detected 3.5 years after the initial gastrectomy. After four cycles of CapeOX combination chemotherapy, the solitary liver lesion was diminished and serum AFP levels decreased to within normal limits. Abbreviations: ACT: adjuvant chemotherapy; AFP: alpha-fetoprotein; CapeOX: capecitabine/oxaliplatin.

**Figure 3:**
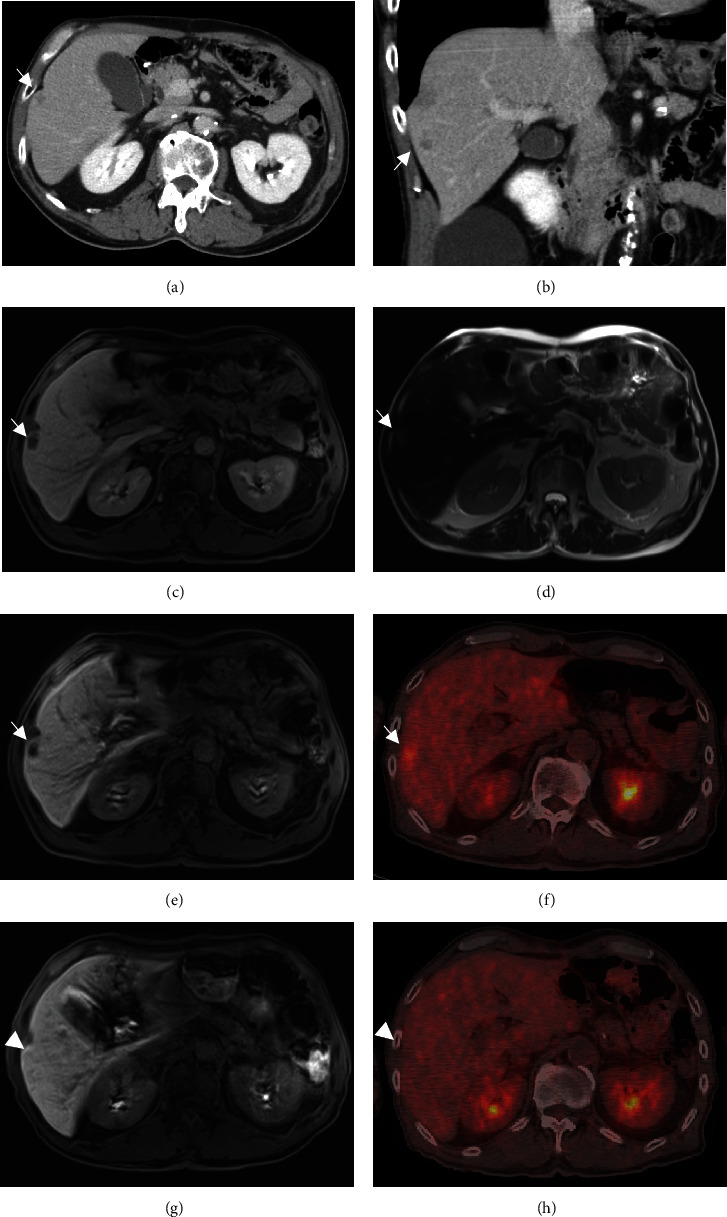
Radiological findings following liver metastasis (segment 5/6) from AFP-producing gastric carcinoma contrast-enhanced computed tomography revealing a hypovascular nodule in segment 5/6 of the liver (a, b). T1- and T2-weighted MRI showing a low-signal nodule and slightly high signal, respectively; a 9 mm defect is observed with EOB-MRI (c–e). ^18^F-FDG PET/CT showing an abnormal accumulation of ^18^F-FDG (maximum standardized uptake value of 4.8) at the same point (f). The tumor was undetectable on EOB-MRI after four courses of CapeOX therapy; no accumulation was detected on PET/CT (g, h). Abbreviations: CapeOX: capecitabine/oxaliplatin; CT: computed tomography; EOB: gadolinium ethoxybenzyl-diethylenetriaminepentaacetic acid; MRI: magnetic resonance imaging; PET: positron emission tomography; ^18^F-FDG: 2-deoxy-2-[fluorine-18] fluoro-D-glucose.

The patient underwent an open total gastrectomy, lymphadenectomy, and Roux-en-Y reconstruction for gastric adenocarcinoma. The histopathological diagnosis was primary gastric cancer (pT2, pN2, M0, and stage IIB (Union for International Cancer Control TNM Classification of Malignant Tumors, 8th edition)) (Figures 1(c)–1(e)). Immunostaining of the primary tumor was positive for AFP and negative for human epidermal growth factor receptor 2 (Figures 1(f) and 1(g)). The patient was treated with eight cycles of S-1 monotherapy (tegafur/gimeracil/oteracil) (120 mg/day on days 1–14, every 3 weeks) as an adjuvant chemotherapy. Serum AFP levels decreased to 3.5 ng/mL (Figure 2), and no recurrence was observed on blood tests every 3 months and CE-CT every 6 months over the 3-year surveillance period. However, serum AFP levels started to increase up to 19.6 ng/mL, 3.5 years after curative gastrectomy (Figure 2). CE-CT revealed a hypovascular nodule in segment 5/6 of the liver (Figures 3(a) and 3(b)). A 9 mm nodule was observed on T1- and T2-weighted MRI; this nodule was categorized as a defect on EOB-MRI (Figures 3(c)–3(e)). Positron emission tomography with 2-deoxy-2-[^18^F] fluoro-D-glucose integrated with computed tomography (^18^F-FDG PET/CT) showed an abnormal accumulation of ^18^F-fluorodeoxyglucose (FDG) (maximum standardized uptake value of 4.8) at the same point (Figure 3(f)). Recurrent liver metastasis from an AFP-producing gastric carcinoma was diagnosed, and the patient subsequently underwent four cycles of CapeOX combination chemotherapy (capecitabine, 3000 mg/day on days 1–14, every 3 weeks; oxaliplatin, 130 mg/m^2^ on day 1, every 3 weeks). No remarkable side effects occurred during the dosing period. The tumor was not detected on CE-CT, EOB-MRI, or contrast-enhanced ultrasonography using perflubutane (CE-US) following treatment; no accumulation of ^18^F-FDG was observed by ^18^F-FDG PET/CT (Figures 3(g) and 3(h)). The level of serum AFP decreased to within the normal range (5.6 ng/mL) (Figure 2). No recurrence was detected at 1.5-year follow-up.

## 3. Discussion

We described a case of metachronous liver metastasis from AFP-producing gastric carcinoma after curative gastrectomy, which resulted in complete resolution of the tumor with CapeOX combination chemotherapy without performing hepatic resection. To the best of our knowledge, no prior studies in the English and Japanese literature have reported the successful treatment of metachronous liver metastasis from AFP-producing gastric carcinoma with CapeOX combination chemotherapy.

The association between primary gastric carcinoma and both high serum AFP levels and liver metastasis was first reported by Bourreille et al. in 1970 [6]. Even early stage AFP-producing gastric carcinomas may be associated with multiple liver metastasis and mortality within a couple of years after curative gastrectomy [7]. Compared to noncurative gastrectomy, curative-intent gastrectomy may improve the 5-year survival rate from 3–42% and the mean survival period from 9–29 months in patients with AFP-producing gastric carcinoma [8]. However, the lower 5-year survival rate in patients with AFP-producing gastric carcinoma compared to those with ordinal gastric carcinoma (5-year survival rate of approximately 70% [3]) suggests that surgery alone may not be adequate for early stage AFP-producing gastric carcinoma.

While a previous study reported the long-term survival of a patient with AFP-producing gastric carcinoma who was treated with an oral derivative of 5-fluorouracil (S-1) monotherapy (a standard postoperative regimen for ordinal gastric carcinoma) [9], our case showed that combined curative gastrectomy and adjuvant S-1 monotherapy were unable to prevent metachronous liver metastasis. A wide range of chemotherapeutic regimens and local treatments have been identified as potentially effective for advanced or recurrent AFP-producing gastric carcinomas; some have been able to achieve complete tumor resolution in the target region or long-term disease-free survival (Table 1) [9–21]. CapeOX combination therapy has been generally administered in cases of gastrointestinal malignancy as a neoadjuvant or conversion therapy; some studies have reported successful treatment outcomes (Table 2) [22–26]. Despite absence of successful treatment of recurrent liver metastasis from AFP-producing gastric carcinoma, we selected CapeOX combination therapy for tumor demonstrating resistance to S-1 monotherapy with reference to a previous report on unresectable AFP-producing hepatoid adenocarcinoma of peritoneum and omentum successfully treated by CapeOX combination therapy [27]. The reason CapeOX combination therapy successfully treated metachronous liver metastasis in the present case is unknown; however, CapeOX combination therapy may have the same potential in treating AFP-producing gastric carcinomas as reported in an *in vivo* study on metastatic colorectal cancer tissue [28], in which the upregulation of thymidine phosphorylase by oxaliplatin might have enhanced the antitumor effect of capecitabine.

According to the latest therapeutic guidelines for metastatic liver tumors, hepatectomy is performed if metachronous liver metastasis from ordinal gastric carcinoma meets the following criteria: (1) R0 resection is achievable, (2) three or fewer in number, (3) smaller than 3–5 cm in size, and (4) disease-free period of >2 years after the initial gastrectomy [29]. While metachronous liver metastasis in the present case satisfied these criteria, we proceeded to administer CapeOX combination therapy with the aim of reducing the risk of postoperative micrometastatic lesions from AFP-producing gastric carcinoma. Fortunately, no metastatic liver tumors have been undetectable on CE-US or PET/CT after chemotherapy, and there have been no signs of recurrence over a 1.5-year surveillance period. Follow-up for the detection of tumor regrowth is ongoing.

Reports on the successful treatment of metachronous liver metastasis from AFP-producing gastric carcinoma with CapeOX chemotherapy in the English and Japanese literature are lacking. At present, there is limited option but to base the use of chemotherapy regimens on guidelines established for ordinal gastric carcinoma [9]. Since AFP-producing gastric carcinoma, which have biological characteristics that are relatively different from those of AFP-producing gastric carcinoma, is a rare subgroup with poor prognosis, we showed the possibility of the useful preoperative option of CapeOX chemotherapy. Additional studies are required to confirm the effectiveness of pre-surgical CapeOX combination therapy in patients with metachronous liver metastasis from AFP-producing gastric carcinoma.

## Figures and Tables

**Table 1:** Case series on multimodal therapy for AFP-producing gastric carcinomas (excluding cases with CapeOX therapy).

Author	Target lesion	Treatment	Chemotherapy regimen	Outcome
Sakurai et al. [10] (Japanese)	Metachronous liver metastasis Portal vein tumor emboli	HAI	HAI − FU + ADM + MMC with UFT (oral)	Alive (disease-free) at 4 years after HAIC

Takahashi et al. [11] (Japanese)	Synchronous liver metastasis	Palliative chemotherapy	5 − FU + LV + ETP + CDDP (FLEP)	CR
	Primary GC	TG	ACT: FLEP and S-1	Alive at 7 years after gastrectomy

Tsuji et al. [12] (Japanese)	Primary GC, lymph node metastasis	NAC	DOC + S − 1 + CDDP	PR
		ACT	S-1	

Amano et al. [13] (Japanese)	Primary GC (HER2 positive) cancerous peritonitis	Palliative chemotherapy	Tratuzumab + DOC + S − 1	PR after 8 cycles

Nishiwada et al. [14] (Japanese)	Primary GC (HER2 positive)	Palliative surgery palliative chemotherapy	Tratuzumab + S − 1 + CDDP, Tratuzumab + DOC + S − 1	Alive at 8 months after chemotherapy initiation (PR)

Koneri et al. [15]	Synchronous liver metastases	Palliative chemotherapy	HAIC − CDDP + S − 1 (oral)	PR
	Metachronous lung metastases	Radiation	—	Transient CR
	Metachronous lung metastases para-aortic lymph node metastasis	Palliative chemotherapy	PTX, DFUR + DOC + CDDP, 5 − FU + ADM + MMC	PD at 54 months after gastrectomy
			Sorafenib	Dead at 60 months after the initial gastrectomy (PR for 2 months and SD for 4 months)

Sun et al. [16]	Primary GC massive lymph node metastases	Palliative chemotherapy	5 − FU + l − OHP + LV, PTX + Capecitabine, S − 1 + l − OHP	PR > gastrectomy Alive at 2 months after gastrectomy (disease-free)

Watanabe et al. [17] (Japanese)	Liver/lung metastasis	Palliative chemotherapy	S − 1 + CDDP (SP), IRI + CDDP	Dead at 15 months after hepatectomy

Akamaru et al. [18] (Japanese)	Primary GC liver metastasis	DG + lateral hepatectomy and ACT	ACT: S − 1 + CDDP (SP)	Alive at 5.5 years after gastrectomy

Doi et al. [19]	Synchronous liver metastases	TACE, HAIC	HAIC − 5 − FU + CDDP	
		Palliative chemotherapy	CDDP + capecitabine	PD
		Palliative chemotherapy	PTX + RAM	Alive at 2 years after recurrence

Miyazaki et al. [20] (Japanese)	Metachronous liver metastasis	Palliative chemotherapy	IRI + S − 1	
			SP	Alive at 10 years after SP therapy

Yasuda et al. [9] (Japanese)	Primary GC	DG and ACT	ACT: S-1	Alive at 5 years after gastrectomy

Ding and Ding [21]	Primary GC (HER2 positive)	Palliative chemotherapy	S − 1 + l − OHP + trastuzumab, DOC + trastuzumab	PD
			Apatinib	PR

Abbreviations: ACT: adjuvant chemotherapy; ADM: adriamycin; AFP: alpha-fetoprotein; CapeOX: capecitabine/oxaliplatin; CDDP: cisplatin; CR: complete response; DFUR: deoxy-5-fluorouridine; DG: distal gastrectomy; DOC: docetaxel; ETP: etoposide; FLEP: 5-fluorouracil; leucovorin; etoposide, and cisplatin; GC: gastric carcinoma; HAI: hepatic arterial injection; HAIC: hepatic artery injection chemotherapy; HER2: human epidermal growth factor receptor 2; IRI: irinotecan; LV: leucovorin; MMC: mitomycin; NAC: neoadjuvant chemotherapy; PD: progressive disease; PR: partial response; PTX: paclitaxel; RAM: ramucirumab; SD: stable disease; SP: tegafur/gimeracil/oteracil and cisplatin; S-1: tegafur/gimeracil/oteracil; TACE: transcatheter arterial chemoembolization; TG: total gastrectomy; UFT: tegafur/uracil; l-OHP: oxaliplatin; 5-FU: 5-fluorouracil. Abbreviations: cited studies, [9–21].

**Table 2:** Summary of previous reports on AFP-producing gastric carcinomas treated with CapeOX combination therapy. The literature review included studies that were published between January 2001 and November 2021. English language articles were searched on the PubMed/MEDLINE database using the terms “hepatoid adenocarcinoma” or “alpha-fetoprotein-producing adenocarcinoma, capecitabine, and oxaliplatin.” A Japanese literature search was conducted using the ICHUSHI (Igaku Chuo Zasshi) database from the Japan Medical Abstracts Society (JAMAS).

Author	Aim of CapeOX	Primary gastric tumor	Liver metastasis	Therapeutic effect	Outcome
Kripp et al. [22]	Palliative	Esophago-gastric junction carcinoma	Synchronous multiple	Long-lasting major remission	Deceased at 18.5 months
Mori et al. [23] (Japanese)	Conversion	AFPGC and rectal cancer	Synchronous multiple	(1) CapeOX therapy	Alive at 29 months
				(2) DG + LAR + partial hepatectomy	(disease-free)
				ACT: not performed	
Fang et al. [24]	Palliative	AFPGC	Synchronous multiple	(1) PTX + LV + 5 − FU (2) Capecitabine (3) TACE − l − OHP + S − 1 (oral) (4) 5 − FU + LV + IRI (5) CapeOX (1 cycle) (6) Sorafenib (oral) (7) nabPTX	Deceased at 30 months after diagnosis due to cholestatic jaundice
Shen et al. [25]	Neo-adjuvant	AFPGC with infiltration into left liver lobe and lymphadenectasis	None	(1) CapeOX therapy	Alive at 7 months after surgery
				(2) Gastrectomy and extended left hepatectomy	
Choi et al. [26]	Palliative (recurrent liver metastasis)	Gastric adenocarcinoma with AFP-positive endodermal sinus tumor component	Metachronous	(1) CapeOX therapy: poor	Deceased at 11 months after BEP
				(2) BEP therapy: partial response and became resectable	Overall survival: 22 months (mortality due to pneumonia)

Abbreviations: ACT: adjuvant chemotherapy; AFP: alpha-fetoprotein; AFPGC: alpha-fetoprotein-producing gastric carcinoma; BEP: bleomycin; etoposide, and cisplatin; CapeOX: capecitabine/oxaliplatin; DG: distal gastrectomy; IRI: irinotecan; LAR: low anterior resection; LV: leucovorin; PTX: paclitaxel; S-1: tegafur/gimeracil/oteracil; TACE: transcatheter arterial chemoembolization; l-OHP: oxaliplatin; nabPTX: nanoparticle albumin-bound paclitaxel; 5-FU, 5-fluorouracil. Abbreviations: cited studies, [22–26].
